# Correction: Efficiency measures the conversion of agonist binding energy into receptor conformational change

**DOI:** 10.1085/jgp.20181221511252019c

**Published:** 2019-12-05

**Authors:** Tapan K. Nayak, Ridhima Vij, Iva Bruhova, Jayasha Shandilya, Anthony Auerbach

Vol. 151, No. 4, April 1, 2019. 10.1085/jgp.201812215.

After publication of this article, several errors were brought to the authors’ attention, which have been corrected as follows, with bold indicating insertions and strikethrough indicating deletions.

Both the HTML and PDF versions of the article have been corrected. These errors appear only in print and PDF versions downloaded on or before December 4, 2019.

In the Results section “Other receptors,” the asterisk in Eq. 5 should have been in the denominator, and some percentages have been adjusted:

“Next, we investigated energy efficiency in other receptors. In terms of equilibrium constants, Eq. 2 is

$$\eta =1-\log({\text{K}}_{\text{dR}})/\log({\text{K}}_{\text{dR}*}),$$ (5)

where K_dR*_ is the equilibrium dissociation constant of the active conformation and K_dR_ is the equilibrium dissociation constant of the resting conformation (Fig. 1 b). For example, K_dR*_ and K_dR_ for ACh measured at the mouse AChR α−ε site are 12 nM and 153 µM (Nayak and Auerbach, 2017), from which we calculate η_ACh_ = **53**52%.

We used Eq. 5 to estimate the efficiency of the agonist Ca^**2+**+2^ at binding sites of KCa1.1 (BK; a potassium-selective ion channel)using published values of the equilibrium dissociation constants (Sweet and Cox, 2008). At Ca-bowl sites, K_dR_ = 3.1 mM and K_dR*_ = 0.9 µM, from which we calculate η_Ca_ = **64**9%. At RCK1 sites, K_dR_ = 15.8 mM and K_dR*_ = 2.1 µM, from which we calculate η_Ca_ = **68**13%.”

In the Discussion section “η and ∆G_0_,” two sentences were deleted:

“Table 5 shows η values for different agonist/site combinations and ΔG_0_ values for seven kinds of receptor. The overall, average

efficiency for the native agonist was ∼51%, with values ranging between 39% (GABAA receptors) and 59% (glycine receptors). Human endplate AChR-binding sites are typical in this regard, with an average efficiency of ∼51%. Apparently, many diverse receptors dedicate about half of the available ligand-binding energy to the activation conformational change. Ca^2+^ at KCa1.1-binding sites is substantially less efficient, for unknown reasons. It is possible that the low per-site efficiency is compensated by the large number of binding sites (n = 8).”

